# Curcumin Enhanced Busulfan-Induced Apoptosis through Downregulating the Expression of Survivin in Leukemia Stem-Like KG1a Cells

**DOI:** 10.1155/2015/630397

**Published:** 2015-10-18

**Authors:** Guangyang Weng, Yingjian Zeng, Jingya Huang, Jiaxin Fan, Kunyuan Guo

**Affiliations:** ^1^Department of Hematology, Zhujiang Hospital, Southern Medical University, Guangzhou 510000, China; ^2^Department of Hematology, Jiangmen Wuyi Traditional Chinese Medicine Hospital, Jiangmen 529000, China; ^3^Department of hemodialysis, Shenzhen Traditional Chinese Medicine Hospital, Shenzhen 518000, China

## Abstract

Leukemia relapse and nonrecurrence mortality (NRM) due to leukemia stem cells (LSCs) represent major problems following hematopoietic stem cell transplantation (HSCT). To eliminate LSCs, the sensitivity of LSCs to chemotherapeutic agents used in conditioning regimens should be enhanced. Curcumin (CUR) has received considerable attention as a result of its anticancer activity in leukemia and solid tumors. In this study, we investigated the cytotoxic effects and underlying mechanisms in leukemia stem-like KG1a cells exposed to busulfan (BUS) and CUR, either alone or in combination. KG1a cells exhibiting BUS-resistance demonstrated by MTT and annexin V/propidium iodide (PI) assays, compared with HL-60 cells. CUR induced cell growth inhibition and apoptosis in KG1a cells. Apoptosis of KG1a cells was significantly enhanced by treatment with CUR+BUS, compared with either agent alone. CUR synergistically enhanced the cytotoxic effect of BUS. Seven apoptosis-related proteins were modulated in CUR- and CUR+BUS-treated cells analyzed by proteins array analysis. Importantly, the antiapoptosis protein survivin was significantly downregulated, especially in combination group. Suppression of survivin with specific inhibitor YM155 significantly increased the susceptibility of KG1a cells to BUS. These results demonstrated that CUR could increase the sensitivity of leukemia stem-like KG1a cells to BUS by downregulating the expression of survivin.

## 1. Introduction

Hematopoietic stem cell transplantation (HSCT) is currently one of the most effective methods of curing hematopoietic malignances [1–3]. In 1977, Thomas reported long-term survival in 13 patients with leukemia who underwent HSCT [4]. However, leukemic patients who received allo-HSCT are still susceptible to relapse and to nonrecurrence mortality (NRM) associated with the toxicity of the chemotherapeutic agents used for conditioning [5, 6], such as busulfan (BUS), cytoxan, and etoposide. Leukemia stem cells (LSCs) are considered to be responsible for leukemia relapse and drug resistance [7, 8]. Complete elimination of LSCs and reduced doses of chemotherapeutic agents are thus essential strategies for improving the prognosis in these patients [9]. Lapidot et al. demonstrated that acute myeloid LSCs possessed the cell phenotype of CD34^+^CD38^−^ [10]. Notably, KG1a cells with a similar phenotype have demonstrated self-renewal potential and chemotherapy and immunotherapy resistance [11, 12]. KG1a cells are thus considered as leukemia stem-like cells and provide an ideal cells model for studying LSCs.

The alkylating agent BUS is commonly applied in different conditioning regimens for HSCT, to eliminate the underlying leukemia cells and exert an immunosuppressive effect. However, BUS is associated with severe toxicities, including liver, lung, and skin toxicities, hemorrhagic cystitis, diarrhea, and mucositis [13, 14]. The ability of BUS to inhibit or effectively kill LSCs also remains unclear, leaving the potential for leukemia relapse after HSCT.

Curcumin (CUR) is a polyphenol derived from the rhizomes of turmeric, which has received considerable attention as a result of its chemopreventive, chemotherapeutic, and chemosensitizing activities in leukemia and various solid tumors, via targeting multiple signaling pathways [15–19]. CUR thus represents a potential sensitizing agent when combined with chemotherapeutic drugs for treating LSCs.

In this study, we explored the cytotoxic efficiencies and molecular mechanisms of CUR and BUS alone and in combination in KG1a cells.

## 2. Materials and Methods

### 2.1. Reagents

Reagents include RPMI-1640 (Hyclone, SH30809.01B), fetal bovine serum (Hyclone, SH30084.03), penicillin and streptomycin (PAA, P11-010), CUR (Sigma, 458-37-7), DMSO (Amresco, 67-68-5), BUS (Sigma, 55-98-1), 3-(4,5-dimethylthiazol-2-yl)-2,5-diphenyltetrazolium bromide (Seebio, 298-93-1), hydroxypropyl methylcellulose (Amresco, 9004-65-3), anti-CD34-PE/CD38-FITC (BD Biosciences, USA), FITC Annexin V Apoptosis Detection Kit I (BD Biosciences, USA), CycleTEST Plus DNA Kit (BD Biosciences, USA), anti-PARP (BD, USA, 1 : 500), anti-caspase-3 (CST, USA, 1 : 5000), anti-survivin (BD, USA, 1 : 5000), ym155 (SELLECK, 781661-94-7), Human Apoptosis Antibody Array Kit (RayBio, USA), electrophoresis apparatus trophoresis (Tanon EPS200), and LI-COR Odyssey Scanner (USA).

### 2.2. Cell Lines and Culture

Human acute myeloid leukemia KG1a cells and human acute promyelocyte leukemia HL-60 cells were cultured in RPMI-1640 with 10% inactivated fetal bovine serum, penicillin, and streptomycin at 37°C under 5% CO_2_, which were kindly presented by Miaorong She (Department of Hematology, Guangdong General Hospital, Guangzhou, China).

### 2.3. Cell Viability Assay

Cells viability was estimated by MTT assay. KG1a and HL-60 cells in logarithmic phase at 5 × 10^5^ cells/mL were incubated in 96-well plates in the presence or absence of the indicated test samples in a final volume of 0.2 mL for 24 h or 48 h at 37°C under 5% CO_2_. 20 *μ*L MTT solution (5 mg/mL in phosphate-buffered saline (PBS)) was then added to each well and incubated for 4 h at 37°C, followed by the addition of 200 *μ*L DMSO. Finally the plates were shaken and examined at 490 nm using a microplate reader (MK3, Shanghai). Each assay was performed in triplicate. Cells viability was calculated as follows: survival ratio (%) = (OD value of experimental samples/OD value of control samples) × 100%.

### 2.4. Flow Cytometry Analysis for Immunophenotyping

Single-cell suspensions of 1.0 × 10^6^ of KG1a and HL-60 cells were washed in PBS containing 2% fetal calf serum (FCS). The cells were resuspended in PBS and incubated for 30 min at 4°C with antibodies to surface antigens CD34 and CD38. Mouse IgG isotype was used as a control. The cells were then analyzed by flow cytometry.

### 2.5. Methylcellulose Colony Formation Test

Approximately 500 treated or untreated cells per well were cultured in RPMI 1640 medium supplemented with 0.9% methylcellulose and 20% fetal bovine serum (FBS) in a final volume of 1 mL at 37°C under 5% CO_2_. Colonies (>50 cells) were counted and photographs were taken under a light microscopy after 14 days. All the samples were analyzed in triplicate.

### 2.6. Measurements of Apoptosis

The apoptotic rates of KG1a and HL-60 cells were determined by annexin V binding assays, according the manufacturer's instructions. Briefly, approximately 1.0 × 10^6^ cells in 6-well plates were treated with various concentrations of the indicated test samples at 37°C under 5% CO_2_ for 48 h. The cells were then harvested to analyze apoptosis. Cells were washed twice with cold PBS and then resuspended in 1x Binding Buffer at a concentration of 1 × 10^6^ cells/mL and 100 *μ*L of the solution (1 × 10^5^ cells) was transferred to a 5 mL culture tube and then 5 *μ*L of FITC annexin V and 5 *μ*L PI were added and the cells were gently vortexed, followed by incubation for 15 min at room temperature (25°C) in the dark. Finally, 400 *μ*L of 1x Binding Buffer was added to each tube and the cells were then analyzed by flow cytometry.

### 2.7. Cell Cycle Analysis

Approximately 1.0 × 10^6^ cells in 6-well plates were treated with various concentrations of the indicated test samples at 37°C under 5% CO_2_ for 48 h. Cell cycle analysis was performed by flow cytometry using the CycleTEST Plus DNA Kit (BD Biosciences), according to manufacture's instructions.

### 2.8. Western Blot Analysis

Total cellular proteins were isolated with lysis buffer (RIPA). Equal amounts of protein were subjected to 10% or 15% polyacrylamide gel electrophoresis and transferred to polyvinylidene difluoride (PVDF) membranes. After blocking with 5% skim milk, the membranes were incubated with primary antibodies (anti-PARP, anti-caspase-3, and anti-survivin) over night at 4°C and then incubated with horseradish peroxidase-conjugated anti-mouse secondary antibody at room temperature for 1-2 h. The protein bands were imaged using a chemiluminescence reagent (CTB, USA) and densities value of the bands was analyzed using Image J software, with glyceraldehyde 3-phosphate dehydrogenase (GAPDH; HC301; 1 : 5000) as the internal reference.

### 2.9. Analysis of Apoptosis-Related Proteins by RayBio Arrays

The expression of 43 apoptosis-related proteins was analyzed using a Human Apoptosis Antibody Array Kit (RayBio, USA). Briefly, according to instructions, each of the capture antibodies was printed on the membranes, followed by addition of the treated or untreated cell lysate. After extensive washing, the membranes were incubated with a cocktail of biotin-conjugated anti-apoptotic protein antibodies. After incubation with the infrared fluorescent agent-streptavidin, the fluorescence signals were visualized using a LI-COR Odyssey Scanner.

### 2.10. Statistical Analysis

The data ware represented as the mean ± standard deviation (SD) and analyzed using SPSS 13.0 and Graphpad Prism 5 software. Means of different groups were compared using one-way ANOVA followed by Bonferroni multiple comparison to evaluate the differences between two groups under multiple conditions. If the date failed the normality test, the Kruskal-Wallis one-way ANOVA on ranks was used for data that failed the normality test. A value of *P* < 0.05 was considered statistically significant. Compusyn software was used to evaluate the synergistic effects of drug combinations. The combination index (CI) was generated by Compusyn software, where CI < 1, CI = 1, and CI > 1 indicated synergism, additive effect, and antagonism, respectively.

## 3. Results

### 3.1. CD34^+^CD38^−^ KG1a Cells Were Insensitive to BUS

**Figure 1:**
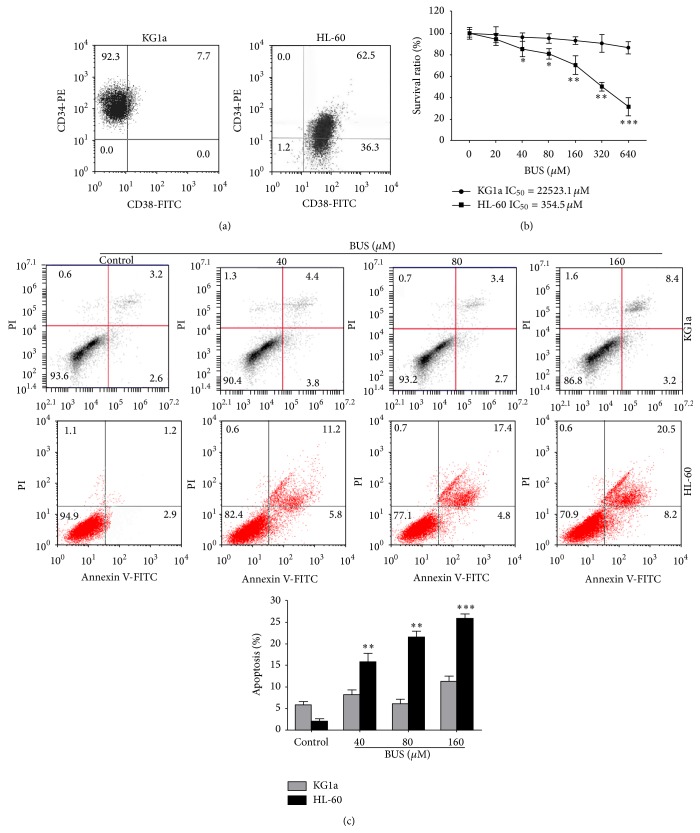
CD34^+^CD38^−^KG1a cells were insensitive to BUS. (a) KG1a cells were stained with FITC-conjugated CD38 antibody and PE-conjugated CD34 antibody and subjected to flow cytometry to analyze the purity of the CD34^+^CD38^−^ cells population. (b, c) KG1a cells were exposed to different concentrations of BUS for 24 or 48 h (c). MTT assay was performed (b) and apoptosis (c) was detected by annexin V/PI assay. Cells in the lower right quadrant represent early apoptosis and cells in the upper right quadrant represent late apoptosis. The graph displays the means ± SD of three independent experiments. ^*∗∗*^ *P* < 0.01, ^*∗∗∗*^ *P* < 0.001 (compared with untreated KG1a cells).

The percentages of CD34^+^CD38^−^ cells were 92.3% in KG1a cells, but no CD34^+^CD38^−^ cells were detected among the HL-60 cells (Figure 1(a)). KG1a and HL-60 cell lines were treated with various concentrations of BUS for 48 h followed by cell viability and apoptosis analyses. BUS suppressed proliferation and induced apoptosis in more mature HL-60 cells, but not in KG1a cells (Figures 1(b) and 1(c)). The IC_50_ values for BUS were 22523.1 *μ*M in KG1a cells and 354.5 *μ*M in HL-60 cells, respectively. The apoptotic rate was significantly higher in HL-60 cells, compared with KG1a cells. These results indicated that leukemia stem-like KG1a cells were insensitive to BUS and exhibited drug resistance.

### 3.2. CUR Inhibited Cell Growth and Induced Cell Apoptosis in KG1a Cells

**Figure 2:**
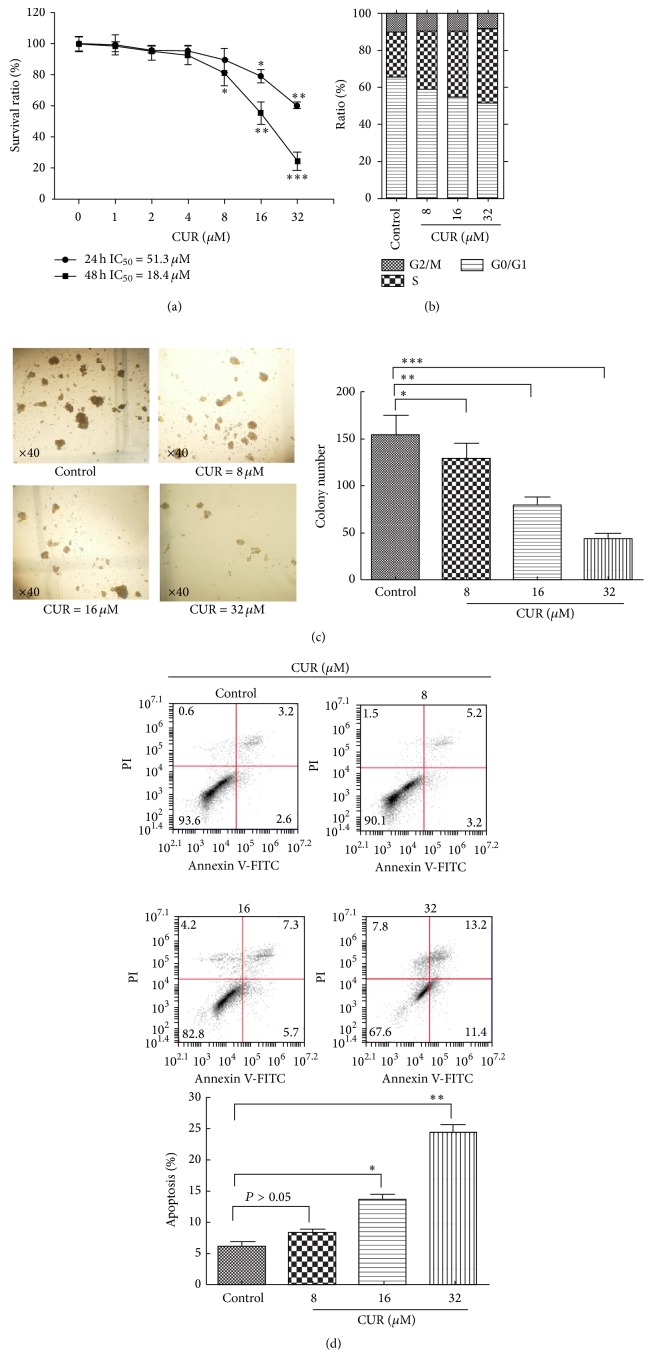
CUR suppressed cell growth, induced S phase arrest, and induced cell apoptosis in KG1a cells. (a) KG1a cells were treated with different concentrations of CUR for 24 or 48 h. MTT assays were performed. (b) KG1a cells were treated with different concentrations of CUR for 48 h and analyzed for DNA content by flow cytometry. (c) KG1a cells were treated with CUR and inoculated in methylcellulose for 14 days and then observed under a right microscope (magnification ×40). The graph displays means ± SD of three independent experiments. ^*∗*^ *P* < 0.05, ^*∗∗*^ *P* < 0.01, and ^*∗∗∗*^ *P* < 0.001 (compared with control). (d) KG1a cells were treated with different concentrations of CUR for 48 h and analyzed by flow cytometry. The graph displays means ± SD of three independent experiments. ^*∗*^ *P* < 0.05, ^*∗∗*^ *P* < 0.01 (compared with control).

KG1a cells were treated with various concentrations of CUR (0–32 *μ*M) for 24 and 48 h and the cytotoxic effects were detected by MTT assay. CUR exhibited dose- and time-dependent cytotoxic effects in KG1a cells (Figure 2(a)). The IC_50_ values at 24 and 48 h were 51.3 *μ*M and 18.4 *μ*M, respectively. The antiproliferation effect of CUR in KG1a cells was confirmed further by colony formation assays. CUR suppressed colony formations in a dose-dependent manner (Figure 2(c)). To determine if CUR-induced growth inhibition was related to the cell cycle arrest, KG1a cells were exposed to CUR for 48 h followed by detection by flow cytometry. CUR induced S phase arrest in KG1a cells (Figure 2(b)). Treatment with 32 *μ*M CUR significantly increased the percentage of cells in S phase from 24.14% to 40.08%. We investigated the effect of CUR for 48 h on early and late apoptosis in KG1a cells by annexin V analysis. CUR induced apoptosis in a dose-dependent manner in KG1a cells (Figure 2(d)). These results demonstrated that CUR could inhibit cell growth and induce apoptosis in KG1a cells.

### 3.3. CUR Increased BUS-Induced Apoptosis by Downregulating Procaspase-3 followed by PARP Degradation in KG1a Cells

**Figure 3:**
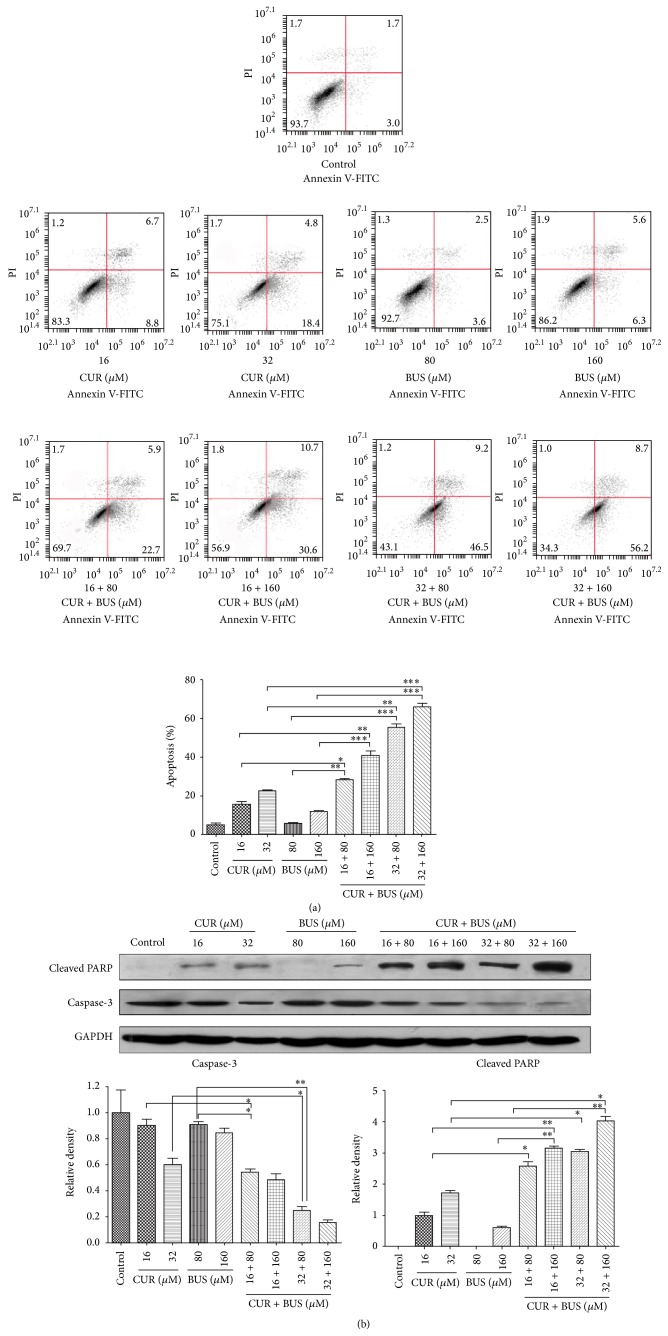
CUR increased BUS-induced apoptosis by downregulating procaspase-3 followed by PARP degradation in KG1a cells. (a, b) KG1a cells were treated with different concentrations of CUR or BUS alone or CUR + BUS for 48 h and analyzed by flow cytometry (a) and western blot (b). The graphs represent means ± SD of three independent experiments. ^*∗*^ *P* < 0.05, ^*∗∗*^ *P* < 0.01, and ^*∗∗∗*^ *P* < 0.001.

We determined if CUR could increase BUS-induced apoptosis in KG1a cells by examining proapoptotic effects of CUR and BUS alone and in combination (CUR + BUS) using annexin V/PI. Apoptosis was significantly increased in CUR + BUS group, compared with CUR- or BUS-alone groups (Figure 3(a)). For instance, apoptotic rates in cells treated with 16 *μ*M CUR, 80 *μ*M BUS, and the combination groups were 15.6 ± 1.5%, 5.7 ± 0.7%, and 28.3 ± 0.8%, respectively. Western blot analysis also demonstrated that the markers of apoptosis procaspase-3 cleaved PARP were significantly regulated in combination groups (Figure 3(b)). These results indicated that CUR significantly enhanced BUS-induced apoptosis.

### 3.4. CUR Synergistically Enhanced the Cytotoxic Effect of BUS in KG1a Cells

**Figure 4:**
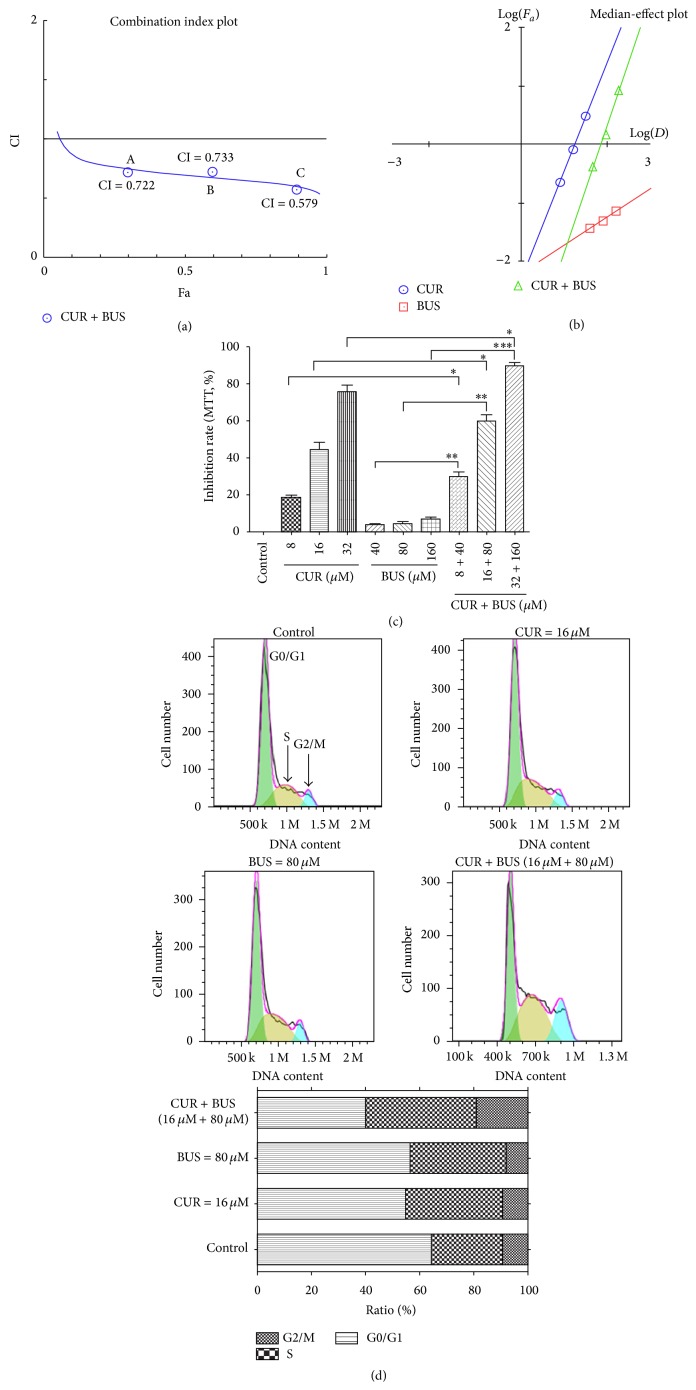
CUR synergistically enhanced the cytotoxic effect of BUS in KG1a cells. KG1a cells were exposed to CUR + BUS at different doses but in a constant ratio (CUR to BUS: 8 *μ*M to 80 *μ*M, 16 *μ*M to 160 *μ*M, and 32 *μ*M to 320 *μ*M, resp.) for 48 h examined by MTT assay. (a, b) CI-effect plots and median-effect plots were generated using Compusyn software. The points A, B, and C represent CI values for the three combination groups, respectively. (c) The graph displays means ± SD of three independent experiments. ^*∗*^ *P* < 0.05, ^*∗∗*^ *P* < 0.01, and ^*∗∗∗*^ *P* < 0.001. (d) KG1a cells were treated with CUR or BUS alone or CUR + BUS for 48 h and analyzed with flow cytometry. The percentages of cells in S and G2/M phases were significantly higher in CUR + BUS group compared with the CUR- or BUS-alone group.

We investigated the ability of CUR to enhance the cytotoxic effect of BUS by treating KG1a cells with combinations of the two drugs at different doses but in a constant ratio (CUR to BUS: 8 *μ*M to 80 *μ*M, 16 *μ*M to 160 *μ*M, and 32 *μ*M to 320 *μ*M, resp.) for 48 h. Synergistic effects were estimated using Compusyn software. Cotreatment with all doses exhibited synergistic effects in KG1a cells (Figures 4(a) and 4(b)). For example, 16 *μ*M CUR plus 80 *μ*M BUS resulted in a proliferation inhibition of 60.20% (Figure 4(c)), compared with CUR (44.40%) and BUS alone (4.53%), indicating a synergistic effect (CI = 0.733), in accord with the result of apoptosis assays. Cotreatment with 16 *μ*M CUR and 80 *μ*M BUS for 48 h also induced S and G2/M phase arrest in KG1a cells (Figure 4(d)), which may represent one of the mechanisms responsible for the synergism.

### 3.5. Effects of BUS and CUR on Protein Expression in KG1a Cells

**Figure 5:**
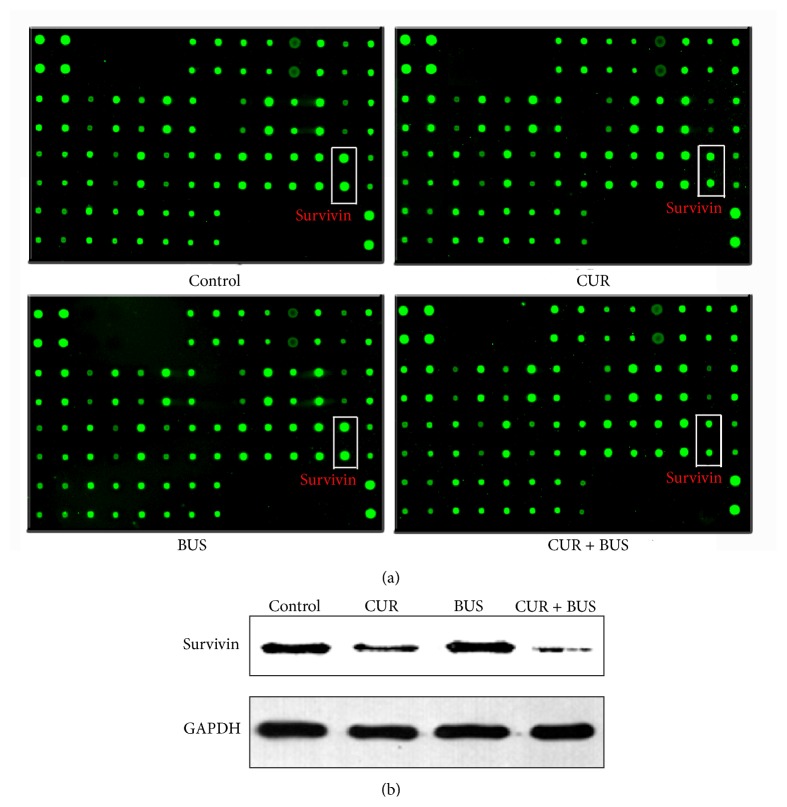
Expression of antiapoptosis protein survivin in KG1a cells. (a, b) KG1a cells were treated with CUR (16 *μ*M), BUS (80 *μ*M), or CUR + BUS for 48 h tested by protein arrays kit (a) as described in “methods.” The intensities of green fluorescence spots represent survivin expression. Survivin expression was significantly decreased in CUR and CUR + BUS groups, compared with controls, the same as the results analyzed by western blot analysis.

We investigated the molecular mechanisms responsible for CUR-induced apoptosis and enhanced BUS-induced apoptosis in KG1a cells treated with 16 *μ*M CUR, 80 *μ*M BUS, and their combination by detecting expression levels of 43 apoptosis-related proteins using RayBio human apoptosis arrays. The threshold values of fold-change were usually set at ≤0.667 or ≥1.5. Three proteins (Bcl-2-associated death promoter (BAD), caspase-3, and HTRA) were upregulated and four proteins (Bcl-2, cellular inhibitor of apoptosis-2 (cIAP-2), survivin, and X-linked inhibitor of apoptosis (XIAP)) were downregulated in CUR group and combination group (Table 1; Figure 5(a)). Survivin was significantly more downregulated in the combination group compared with the CUR group. This result was further confirmed by western blot analysis (Figure 5(b)). Survivin is known to be an important antiapoptosis protein that participates in the modulation of apoptosis by various signal pathways. We therefore considered that survivin was a likely key factor in CUR-induced apoptosis and BUS sensitivity in KG1a cells.

### 3.6. Suppression of Survivin with YM155 Could Induce Apoptosis and Increase the Susceptibility to BUS in KG1a Cells

**Figure 6:**
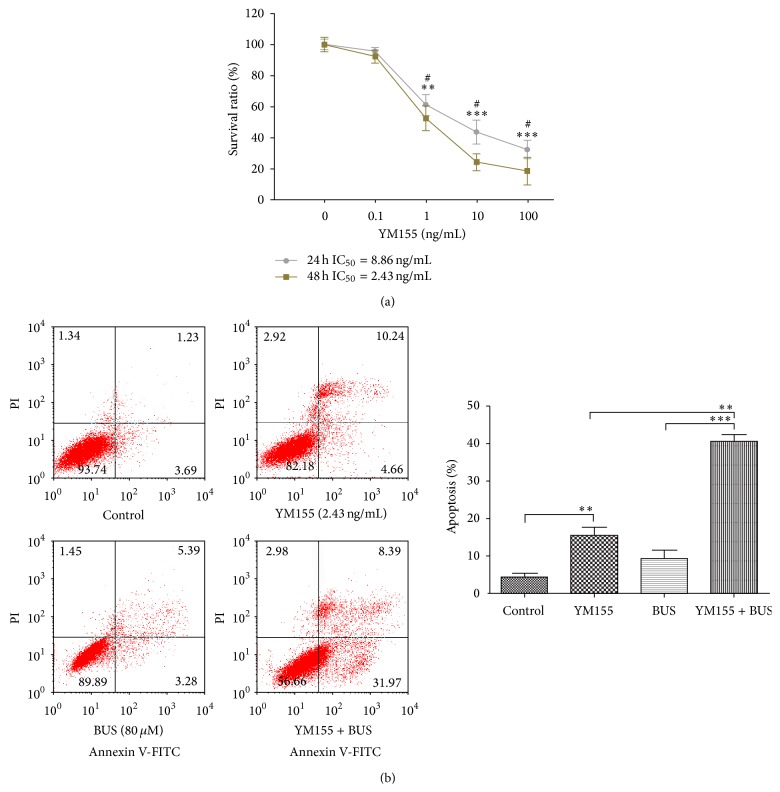
Suppression of survivin with YM155 could induce apoptosis and increase the sensitivity to BUS in KG1a cells. (a) KG1a cells were treated with different concentrations of YM155 for 24 and 48 h and examined by MTT assay. ^*∗∗*^ *P* < 0.01 and ^*∗∗∗*^ *P* < 0.001 (compared with control) and ^#^ *P* < 0.05 (compared with 48 h group). (b) KG1a cells exposed to YM155 (2.43 ng/mL) and BUS (80 *μ*M) alone or CUR + BUS were analyzed by flow cytometry. The graph displays means ± SD of three independent experiments. ^*∗∗*^ *P* < 0.01, ^*∗∗∗*^ *P* < 0.001.

We clarified the role of CUR-induced survivin downregulation in sensitization of KG1a cells to BUS by suppressing survivin expression using the specific inhibitor YM155. The proapoptotic effect and sensitivity to BUS were evaluated by flow cytometry. The cytotoxic activity of YM155 in KG1a cells was detected by MTT assays. YM155 exhibited time- and dose-dependent growth-inhibitory effects in KG1a cells (Figure 6(a)). The IC_50_ values of 24 and 48 h were 8.86 ng/mL and 2.43 ng/mL, respectively. The YM155 IC_50_ of 2.43 ng/mL was used in subsequent experiments. KG1a cells were exposed to 2.43 ng/mL YM155 and 80 *μ*M BUS alone or in combination for 48 h and early and late apoptotic rates were then examined. YM155-induced apoptosis (14.90%) (Figure 6(b)) was similar to CUR-induced apoptosis in KG1a cells (15.50%, 16 *μ*M, Figure 3(a)). Suppression of survivin by YM155 increased the susceptibility to BUS, with a BUS-induced apoptotic rate of 40.36%, compared with 8.67% for BUS alone. These results revealed that suppression of survivin could contribute to CUR-induced apoptosis and the synergistic effect of CUR and BUS in KG1a cells.

## 4. Discussion

LSCs were a rare population of cells in patients with leukemia. They possess characteristics of self-renewal, chemotherapy resistance, and immune resistance [20–22]. LSCs were thus commonly regarded as the origin of leukemia relapse and refractory [12, 23]. LSCs have been reported to demonstrate a CD34^+^CD38^−^ phenotype [10, 12, 24, 25], reflected by the acute immature myeloid leukemia cells KG1a cell line, which expresses high level of CD34 and lacks CD38. We also provided the first demonstration that leukemia stem-like KG1 cells were insensitive to BUS according to MTT assays and annexin V/PI assays, compared with the more mature acute promyelocyte leukemia HL-60 cells. KG1a cells have previously been shown to be resistant to the common chemotherapeutic agent daunorubicin [12]. CD34^+^CD38^−^ KG1a cells maybe thus provide an ideal model of LSCs, in accord with previous studies [12, 26].

CUR and its analogs have been showed to suppress the growth of various leukemia cells, including U937 cells [27, 28], K562 chronic myeloid leukemia cells [27], and HL-60 acute promyelocyte leukemia cells [29, 30], but its effects on LSCs have not been determined. CUR inhibited proliferation and induced S phase arrest and apoptosis in leukemia stem-like KG1a cells. CUR was previously shown to target cancer cells or cancer stem cells by several mechanisms, including autophagy, G2/M phase arrest, and apoptosis in hepatoma cells (HepG2, SMMC-7721, and BEL-7402) [31], reducing the expression of stem cell markers (DCLK1/Lgr5/CD44) in colon cancer stem-like HCT-116 [32], and reducing microtentacles and preventing reattachment in breast cancer stem-like cells [33]. CUR has thus demonstrated indeed extensive anticancer effects in various tumors and has been shown to modulate numerous targets including the activation of transcription factors (NF-kB, STAT3, and AP-1), receptors (CXCR-4, HER-2, and IL-8), kinases (EGFR, ERK, and JAK), cytokines (TNF, IL), and others (cyclin-D1/E,XIAP-1)[15, 34]. Unfortunately, the mechanism of S phase arrest induced by CUR was not explored further in depth in this study. In a word, CUR exhibited an inhibitory effect on leukemia stem-like KG1a cells, which was particularly worthy of attention.

Insensitivity of LSCs to conditioning chemotherapeutic drugs such as BUS is a major reason for leukemia relapse after HSCT. In this study, KG1a cells displayed resistance to BUS, indicated by a lack of apoptosis induction. We there explored the effects of the combination of CUR and BUS on apoptosis in KG1a cells. Encouragingly, CUR markedly enhanced BUS-induced apoptosis, as confirmed by annexin V/PI and western blot analysis. Similarly, the combination of various concentrations of CUR and BUS produced a synergistic antiproliferation effect in KG1a cells. Accumulating evidence suggests that CUR potentiates the effect, including enhancing the antiapoptotic effects of chemotherapeutic drugs such as 5-fluorouracil, bortezomib, FOLFOX, and paclitaxel* in vitro* or* in vivo* [35–39]. The results of the current study suggested that CUR has the potential to be a powerful chemosensitizing agent in various cancer cells, including cancer stem cells (CSCs). Notably, Yu et al. demonstrated that CUR either alone or together with FOLFOX could efficiently eliminate FOLFOX-resistant colon cancer stem cells [36]. However, the effects of the combination of CUR with BUS on cancer stem cells, especially LSCs, have not been reported. BUS is well-known conditioning agent for HSCT, and its ability to eliminate LSCs is vital for the successful cure of leukemia in patients undergoing this treatment. Gerber et al. pointed out that minimal residual disease detected during complete remission was enriched for CD34^+^CD38^−^ALDH^int^ leukemia cells, which were highly correlated with subsequent clinical relapse [25]. Combined treatment with CUR may allow a reduction in the clinical dose of BUS for HSCT, with the potential for reducing NRM. Nakane et al. showed that reduced-intensity conditioning by BUS was associated with lower NRM in patients undergoing unrelated bone marrow transplantation [40]. The results of the current study showed a significant reduction in the percentage of cells in G0/G1 phase in the combination group (Figure 4(d)), suggesting that cells in G0/G1 phase were more sensitive to this drug combination. Interestingly, cancer stem cells (including LSCs) tend to remain in quiescent phase and possess drug resistance [41–44]. The discovery that CUR could sensitize leukemia stem-like KG1a cells to BUS suggested that further studies are warranted, especially with a view to elucidating the mechanism responsible for this effect.

**Figure 7:**
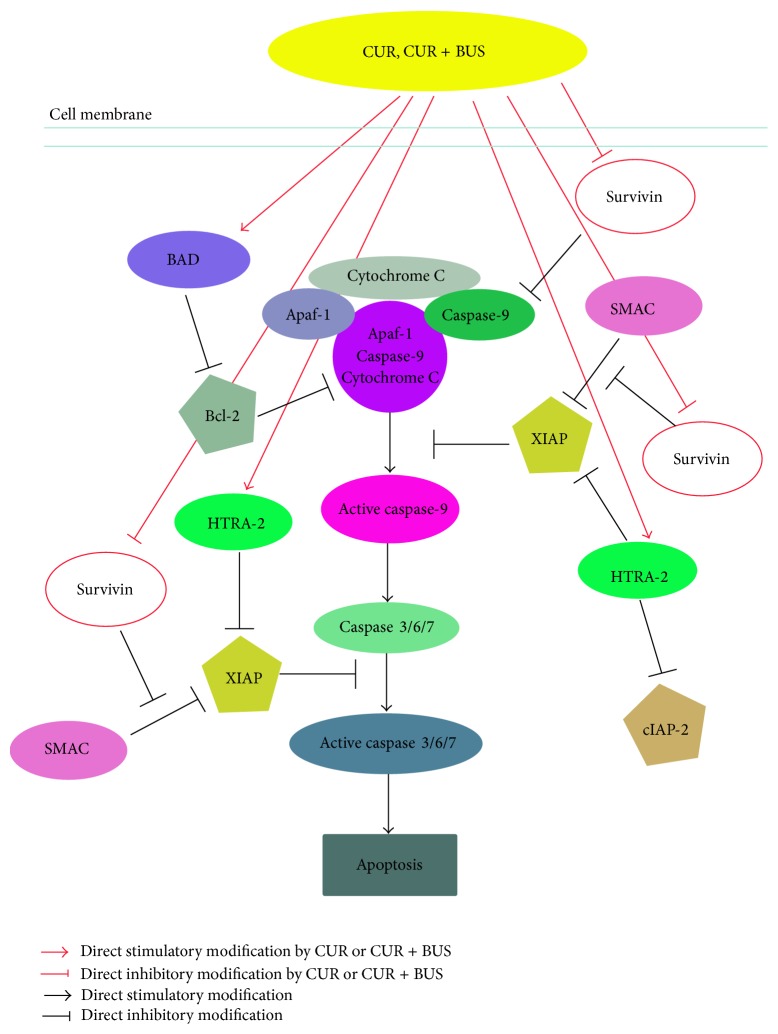
Mechanisms of CUR-induced apoptosis and enhanced sensitivity to BUS in KG1a cells, indicating the potential role of survivin.

The results of apoptosis arrays showed that seven apoptosis-related proteins were significantly modulated in KG1a cells treated with CUR and CUR + BUS (Figure 5(a); Table 1). A mechanistic diagram was thus presented in Figure 7. Activated caspase-3 is the common effector caspase of the intrinsic and extrinsic pathways of apoptosis and is thus a marker of apoptosis [45]. Activated caspase-9 is an upstream protein effector that may stimulate caspase-3 [45]. XIAP inhibits caspases, including caspase-3 and caspase-9, by direct physical interactions [46]. Interestingly, we found that XIAP expression in KG1a cells was downregulated by CUR and especially by CUR + BUS. cIAP-2, another member of inhibitor of apoptosis (IAP) family, was downregulated in the same two groups. cIAP-2 could bind caspase-3 and mark it for proteasomal degradation rather than inhibit it by physical interaction [47]. These results suggest that the downregulation of XIAP and cIAP-2 was closely related to the CUR-induced enhancement of apoptosis in KG1a cells. Notably, we provide the first evidence to demonstrate that CUR alone, and especially in combination with BUS, increased the expression of proapoptotic serine protease HTRA-2 in leukemia cells, particularly in leukemia stem-like cells (Table 1). HTRA2 plays a pivotal role in the induction of apoptosis in the response to various stressors, mediating interactions with a variety of inhibiter of IAPs, such as XIAP and cIAP-1/2, through their BIR domains [48–50]. The neutralization of IAPs causes the activation of caspases 3/7/9 and thus contributes to the induction of apoptosis [48, 49]. Hence, the increase in HTRA-2 observed in the current study may thus be an important mechanism in the downregulation of XIAP and cIAP-2, finally, leading to apoptosis induction and enhancement of apoptosis in CUR + BUS-treated KG1a cells.

This study also demonstrated that survivin expression was downregulated by CUR and CUR + BUS (Figures 5(a) and 5(b); Table 1). Survivin is an important IAP that tends to be overexpressed in cancer cells [51], including cancer stem cells [52, 53], which exerts antiapoptotic effects via various mechanisms. For example, survivin inhibits caspase-dependent apoptosis through cooperation with XIAP, inhibits the SMAC-XIAP complex, and interferes with caspase-3/caspase-9 [51] (Figure 7). Our results showed that KG1a cells overexpressed survivin protein (Figure 5(a)), in accord with the characteristics of leukemia stem-like cells. CUR alone and especially CUR + BUS decreased survivin expression in KG1a cells (consistent with the results of apoptosis showed in Figures 3(a) and 3(b)). Growing evidence has demonstrated that downregulating or inhibiting survivin could induce apoptosis and eradicate cancer stem cells or LSCs [52, 54–56]. This suggests that CUR may induce apoptosis and enhance BUS-induced apoptosis by downregulating the expression of survivin in KG1a cells. This was confirmed by treating KG1a cells with survivin inhibitor YM155 alone or in combination with BUS. YM155 induced apoptosis and enhanced BUS-induced apoptosis in KG1a cells, in a similar manner to CUR (Figure 6(b)). Survivin appears to act as a key protein in the mechanisms whereby CUR sensitizes KG1a cells to BUS. BAD and Bcl-2 proteins were also shown to be modulated by CUR and CUR + BUS, and further studies are warranted to explore their roles in the CUR-induced effects in KG1a cells.

In summary, this study demonstrated underlying new mechanisms whereby CUR may overcome BUS insensitivity by downregulating survivin in leukemia stem-like KG1a cells. CUR, alone or in combination with BUS, could be a potential anti-LSCs agent for preventing leukemia relapse and reducing the NRM after HSCT. BUS is currently still widely used in the pretreatment of HSCT, but it shows significant side effects and carcinogenicity in patients undergoing HSCT, resulting in danger of being replaced by other conditioning regimens. CUR may solve these issues by combining BUS in the conditioning regimen.

## Figures and Tables

**Table 1:** Expression of apoptosis-related proteins in various treated groups.

Name	Control	CUR	BUS	CUR + BUS	CUR/control	BUS/control	CUR + BUS/control
					(fold-change)	(fold-change)	(fold-change)
**BAD**	**9754.372**	**11939.256**	**8854.383**	**21865.952**	**1.224**	**0.908**	**2.242**
BAX	11665.057	11534.453	11072.484	10875.680	0.989	0.949	0.932
**Bcl-2**	**5201.059**	**4032.165**	**4793.070**	**2454.999**	**0.775**	**0.922**	**0.472**
Bcl-w	3934.754	3354.182	3464.131	3789.188	0.852	0.880	0.963
BID	1241.608	1159.421	1473.127	1702.301	0.934	1.186	1.371
BIM	8791.171	7763.556	8225.960	8295.705	0.883	0.936	0.944
**Caspase-3**	**5414.356**	**9883.863**	**4966.096**	**12514.137**	**1.825**	**0.917**	**2.311**
Caspase-8	6636.880	7681.166	7505.017	7338.462	1.157	1.131	1.106
CD40	7229.618	5707.767	6661.514	5874.868	0.789	0.921	0.813
CD40L	16087.024	12956.135	15884.779	14392.403	0.805	0.987	0.895
**cIAP-2**	**1971.306**	**1308.320**	**1917.709**	**702.301**	**0.664**	**0.973**	**0.356**
CytoC	8302.835	6758.989	8156.269	7832.756	0.814	0.982	0.943
DR6	3370.080	2669.250	3008.736	2958.532	0.792	0.893	0.878
Fas	25854.867	22694.082	24157.601	23693.574	0.878	0.934	0.916
FasL	7155.526	6020.454	7420.907	7047.913	0.841	1.037	0.985
HSP27	2395.653	2109.392	2279.382	2807.833	0.881	0.951	1.172
HSP60	24408.943	20936.090	30040.496	23881.647	0.858	1.231	0.978
HSP70	6055.367	7532.267	6146.040	6971.961	1.244	1.015	1.151
**HTRA**	**10052.830**	**20991.713**	**12382.184**	**26027.247**	**2.088**	**1.232**	**2.589**
IGF-I	1772.604	1407.585	1857.630	1717.974	0.794	1.048	0.969
IGF-II	7991.872	8066.316	10003.085	9495.273	1.009	1.252	1.188
IGFBP-1	3060.239	2144.135	2680.707	2416.014	0.701	0.876	0.789
IGFBP-2	3750.645	2977.966	3901.504	3569.769	0.794	1.040	0.952
IGFBP-3	5321.179	3985.511	5547.657	5313.061	0.749	1.043	0.998
IGFBP-4	2012.843	1573.358	1736.271	1873.496	0.782	0.863	0.931
IGFBP-5	11366.442	9202.906	10929.497	10428.404	0.810	0.962	0.917
IGFBP-6	2354.116	1950.567	2340.662	2413.603	0.829	0.994	1.025
IGF-1sR	5755.629	4286.285	5791.576	5056.269	0.745	1.006	0.878
Livin	7478.838	6829.468	7818.628	7778.504	0.913	1.045	1.040
p21	17207.390	15850.718	18463.352	17993.517	0.921	1.073	1.046
p27	8486.943	7790.358	8890.430	8879.213	0.918	1.048	1.046
p53	9829.587	9303.164	11354.853	11263.882	0.946	1.155	1.146
SMAC	9838.568	10157.840	11915.987	12549.047	1.032	1.211	1.275
**Survivin**	**76507.100**	**31134.629**	**81877.505**	**11691.497**	**0.407**	**1.070**	**0.153**
sTNF-R1	3284.761	2747.670	3781.346	3136.960	0.836	1.151	0.955
sTNF-R2	3504.793	2428.035	3118.079	2815.066	0.693	0.890	0.803
TNF-alpha	2641.505	1771.889	2802.065	2499.200	0.671	1.061	0.946
TNF-beta	6946.720	4871.952	6715.585	6430.648	0.701	0.967	0.926
TRAILR-1	3835.964	3031.569	4114.182	4102.643	0.790	1.073	1.070
TRAILR-2	7488.942	6315.272	7505.017	7691.701	0.843	1.002	1.027
TRAILR-3	4649.857	3717.494	4614.036	4605.376	0.799	0.992	0.990
TRAILR-4	4613.933	3900.142	4694.541	4706.646	0.845	1.017	1.020
**XIAP**	**5465.996**	**2412.352**	**6570.195**	**1011.597**	**0.441**	**1.202**	**0.185**

KG1a cells were treated with CUR (16 *μ*M), BUS (80 *μ*M) alone, or CUR + BUS for 48 h tested by protein arrays kit. The data represent fluorescence intensities of 43 apoptosis-related proteins. The bold bands indicate proteins that were modulated by CUR or CUR + BUS. The threshold values of fold-change were usually set at ≤0.667 or ≥1.5.
